# Separation Process of Fine Coals by Ultrasonic Vibration Gas-Solid Fluidized Bed

**DOI:** 10.1155/2017/4763937

**Published:** 2017-08-06

**Authors:** Shuai Wang, Yaqun He, Hua Wei, Weining Xie

**Affiliations:** ^1^Advanced Analysis and Computation Center, China University of Mining and Technology, Xuzhou 221116, China; ^2^School of Chemical Engineering and Technology, China University of Mining and Technology, Xuzhou, Jiangsu 221116, China

## Abstract

Ultrasonic vibration gas-solid fluidized bed was proposed and introduced to separate fine coals (0.5–0.125 mm fraction). Several technological methods such as XRF, XRD, XPS, and EPMA were used to study the composition of heavy products to evaluate the separation effect. Results show that the ultrasonic vibration force field strengthens the particle separation process based on density when the vibration frequency is 35 kHz and the fluidization number is 1.8. The ash difference between the light and heavy products and the recovery of combustible material obtain the maximum values of 47.30% and 89.59%, respectively. The sulfur content of the heavy product reaches the maximum value of 6.78%. Chemical state analysis of sulfur shows that organic sulfur (-C-S-), sulfate-sulfur (-SO_4_), and pyrite-sulfur (-S_2_) are confirmed in the original coal and heavy product. Organic sulfur (-C-S-) is mainly concentrated in the light product, and pyrite-sulfur (-S_2_) is significantly enriched in the heavy product. The element composition, phase composition, backscatter imagery, and surface distribution of elements for heavy product show concentration of high-density minerals including pyrite, quartz, and kaolinite. Some harmful elements such as F, Pb, and As are also concentrated in the heavy product.

## 1. Introduction

Coal is an important primary energy source worldwide, especially in China. In China, coal-fired power is predominant in the production of the Chinese electric power and accounts for more than 70% [1]. Coal contains appreciable quantity of inorganic minerals and harmful elements, such as sulfur, lead, arsenic, and mercury; these minerals and elements can be transformed into inhalable particles, acid rain, and other pollutants and can be discharged into the atmosphere during coal combustion [2], thereby resulting in serious pollution to the atmosphere and large economic losses [3–6]. Thus, desulfurization and deashing of coal prior to combustion are important to prevent fog haze weather.

Although lump coals are separated prior to milling to remove large pieces of waste rock, fine waste rocks containing harmful elements inlay in the coal are not always removed. If coal is sufficiently crushed to fine particles, then the mineral particles can be fully dissociated from the coal. Such condition is favorable to the separation process. Fine coals are currently separated mainly by flotation, which can effectively separate <0.5 mm fraction coal [7–9]. The development of cyclonic-static microbubble flotation column and new reagent systems has enabled good results of low-rank coal separation [9–13]. However, the flotation process consumes large amounts of water, and its development in arid regions is limited by water resource deficiency. Thus, high-efficiency dry separation technology of fine coals should be investigated.

Dry separation technologies, especially the separation technology of the gas-solid fluidized bed, are currently used to separate coal [14–19]. For example, air dense medium fluidized bed is used to effectively separate coal of 50−6 mm size fraction [20–24]. Xu and Zhu [25] examined the influence of vibration parameter on the fluidization characteristics of fine materials. Luo et al. [26] separated coal of 6−1 mm size fraction by use of air dense medium fluidized bed and analyze the particle force condition. Yang et al. [27, 28] used vibrated fluidized bed to separate coal of 6−3 and 3−1 mm size fractions without a dense medium. These abovementioned dry separation methods can effectively separate >0.5 mm fraction coal but present difficulty in separating <0.5 mm fraction coal and exhibit many limitations. Thus, new dry separation methods to deal with fine coal of <0.5 mm fraction should be explored.

This study investigated the separation process of the 0.5−0.125 mm fraction coal with ultrasonic vibration gas-solid fluidized bed. The composition of products under different experimental conditions was studied by advanced analysis and test methods to evaluate the separation results.

## 2. Materials and Methods 

### 2.1. Sampling and Experimental Device

**Figure 1:**
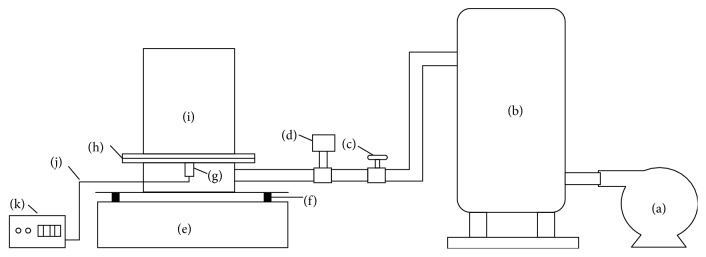
Schematic diagram of the experimental system: (a) roots blower; (b) air reservoir; (c) air valve; (d) vortex flow meter; (e) pedestal; (f) spring; (g) ultrasonic transducer; (h) air distribution plate; (i) fluidized bed; (j) cable; (k) ultrasonic power supply.

The 0.5−0.125 mm fraction coal was chosen to study the separation process. The ash content of the coal was 36.21%, and its sulfur content reached 2.82%. The coal was obviously of high sulfur content. The schematic of experimental system is shown in Figure 1(). The system included air supply and separation systems. The air supply system included a roots blower, an air reservoir, a rotor flow meter, and an air valve. The separation system included a gas-solid fluidized bed and an ultrasonic vibration device. The fluidized bed was made of organic glass (radius of 75 mm and height of 300 mm), and the ultrasonic vibration device included one ultrasonic transducer and one ultrasonic power supply. The ultrasonic transducer was fixed to the bottom of the air distribution plate. The air came from the blower and enters the fluidized bed through the pipe, air reservoir, rotor flow meter, and air distributor. The vibration force field came from the ultrasonic vibrator, which was controlled by ultrasonic generator. The height of the static bed containing the material was 100 mm. The product was divided into five layers, and the thickness ratio of each layer from upper to lower was 1 : 1 : 1 : 1 : 1.

To obtain high-density mineral as pure as possible, the product of the fifth layer was collected as the heavy product, whereas that in the upper four layers was collected as the light product. The ash contents of light and heavy products were measured. The recovery of combustible material was calculated using (1) to estimate the separation effect.

(1) $$\begin{array}{c} E=\gamma_{j}\times \left(\frac{100-{Ad}_{j}}{100-{Ad}_{y}}\right)\times 100\%, \end{array}$$

where *E* is the recovery of combustible material; *γ*_*j*_ is the yield of light product; Ad_*j*_ and Ad_*y*_ are the ash contents of the light product and raw coal, respectively.

### 2.2. XRF Analysis

X-ray fluorescence spectrometer (XRF, S8 Tiger, Bruker, Germany) was applied to the sulfur content analysis for different products to study the separation effect at different experimental conditions. The XRF worked at 20 kV–60 kV and 10 mA–100 mA; the collimator angle was 0.23°.

### 2.3. XRD Analysis

The phase composition analysis was run with an X-ray diffractometer (XRD, D8 Advance, Bruker, Germany) for heavy product from separation process with a voltage of 40 kV and a current of 30 mA. XRD data were recorded in a scanning mode from the detective angle of 3°–90° with the step of 0.01945° (step) and the scanning speed of 0.1 s/step.

### 2.4. XPS Analysis

X-ray photoelectron spectrometer (XPS, ESCALAB 250Xi, Thermo Fisher, America) with Al Ka radiation (hv = 1486.6 eV) and a 900 *μ*m light spot size was used to analyze the chemical state of sulfur in the coal.

### 2.5. EPMA Analysis

Field emission electron probe microanalyzer (EPMA, 8050G, Shimadzu, Japan) was applied to the microstructure, backscatter imaging, and area distribution of element analysis for the heavy products. The beam size was Min, the BC electric current was 10−100 nA, and the testing voltage was 15 kV.

## 3. Results and Discussion

### 3.1. Effect of Operating Parameters on Separation

**Figure 2:**
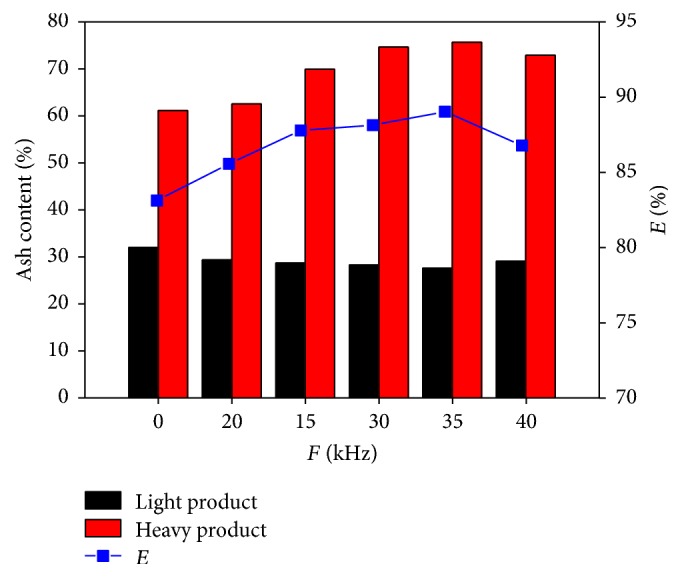
Separation results at different vibration frequency.

The separation results in Figure 2() present the influence of vibration frequency ranging from 20 kHz to 40 kHz and no vibration at a fluidization number of 1.6 and a separation time of 30 s. The ash difference between the light and heavy products and the recovery of combustible material are all minimal at 29.08% and 83.13%, respectively, under no vibration condition. With the increase in vibration frequency from 20 kHz to 35 kHz, the ash difference between the light and heavy products and the recovery of combustible material increase and reach the maximum values of 47.07% and 89.04%, respectively, at 35 kHz. After 35 kHz, the ash difference and the recovery of combustible material decrease. Thus, the addition of ultrasonic vibration field intensifies the separation process of fine coal under different densities.

Table 1 shows the sulfur content of each layer of product at different vibration frequencies. Sulfur is mainly concentrated in the fifth layer, the contents of which are all above 6%, and the maximum value is 6.89% when the vibration frequency is 35 kHz. On the contrary, the sulfur contents of the first layer at different vibration frequencies are low at below 1.6%. The minimum value is 1.26% when the vibration frequency is 35 kHz.

**Figure 3:**
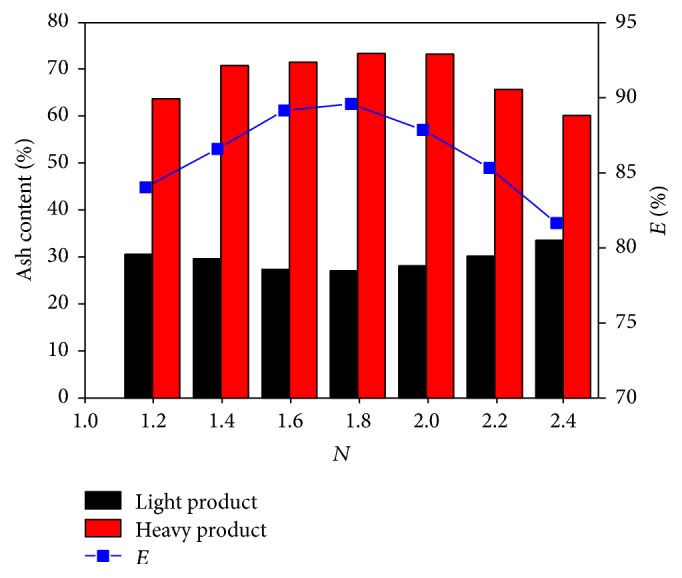
Separation results at different fluidization number.

Figure 3() shows the separation results obtained at different fluidization numbers at a vibration frequency of 35 kHz and at a fluidizing time of 30 s. At low fluidization number, the bed liquidity is poor and the resistance to particle sedimentation is high. Thus, the separation of the coal and high-density minerals becomes difficult. With the increase in the fluidization number, the bed fluidity and the separation effect also increase. The ash difference between the light and heavy products and the recovery of combustible material obtain the maximum values of 47.30% and 89.59%, respectively, when the fluidization number is 1.8. Thereafter, the separation effect decreases with the further increase in the fluidization number. The reason is that the bed stability is destroyed because of the increase in gas velocity, thereby leading to serious back mixing between the light and heavy products.

Table 2 shows the sulfur content of each layer of product at different fluidization numbers. Similarly, sulfur is mainly concentrated in the fifth layer, and the maximum value is 6.78% when the fluidization number is 1.8. The sulfur contents of the first layer are all below 2.0%, and the minimum value is 1.22% when the fluidization number is 2.0.

### 3.2. Component Analysis of Products

The results of the main element content analysis of the original coal and light and heavy products are shown in Table 3. In the light product, the contents of magnesium, calcium, iron, silicon, aluminum, and sulfur decrease compared with those in the original coal. On the contrary, these contents obviously increase in the heavy product, especially those of sulfur and iron.

**Figure 4:**
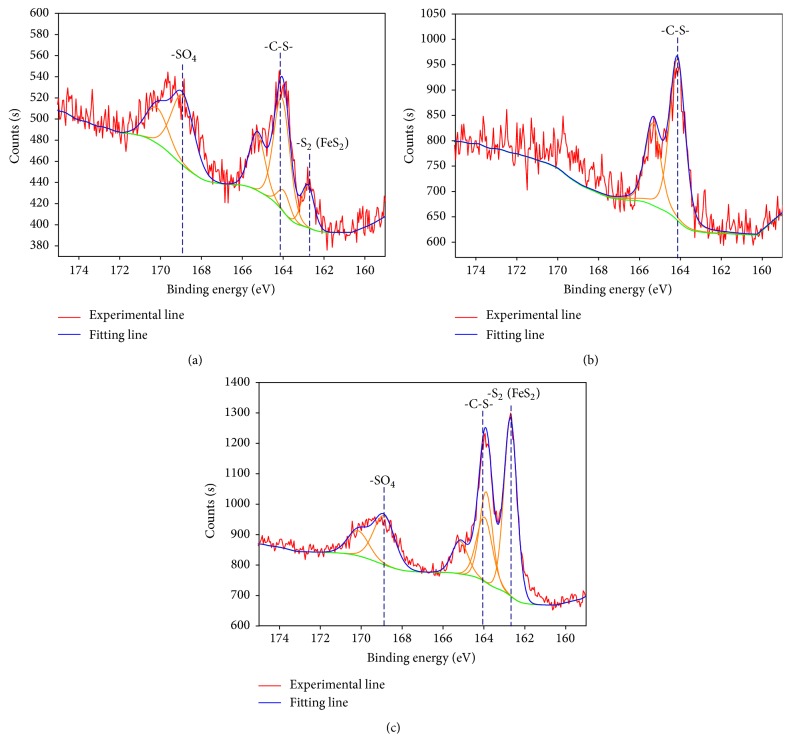
X-ray photoelectron spectroscopy spectra of the coal: (a) original coal; (b) light product; (c) heavy product.

The XPS spectra of sulfur in different samples are shown in Figure 4(), and the content of sulfur in different chemical states is shown in Table 4. Organic sulfur (-C-S-), sulfate-sulfur (-SO_4_), and pyrite-sulfur (-S_2_) are confirmed in the original coal and heavy product by their binding energies, whereas mainly organic sulfur (-C-S-) exists in the light product. In the original coal, the contents of -SO_4_, -C-S-, and -S_2_ are 40.75, 45.75, and 13.5 at.%, respectively. The contents are 24.03, 23.43, and 52.54 at.% in the heavy product. According to the peak area, the pyrite and sulfate are evidently enriched in the heavy product.

**Figure 5:**
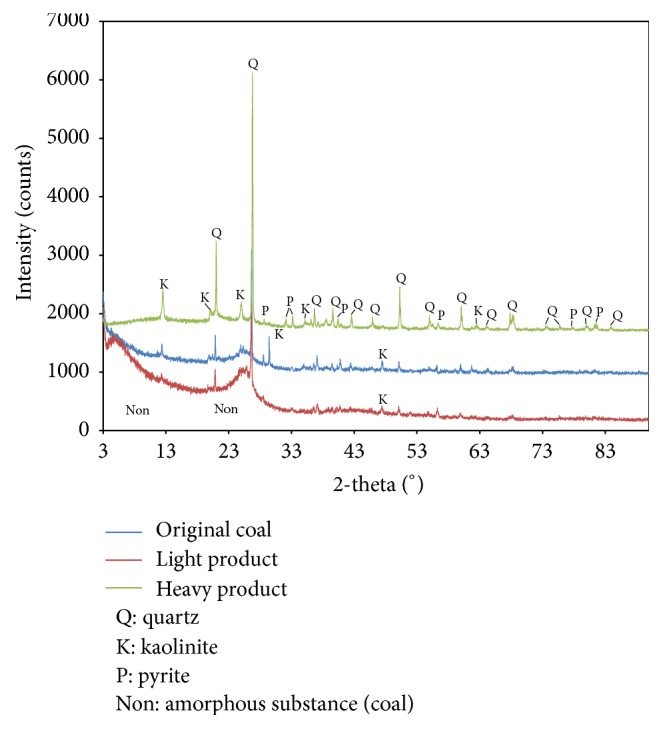
X-ray diffraction of the different samples.

Figure 5() shows the XRD analysis results for the original coal and light and heavy products. The wide and dispersion diffraction peaks of the light product show obvious amorphous characteristics, and the original coal exhibits a few sharp diffraction peaks of mineral. At the same time, the heavy product presents obvious crystal characteristics due to the appearance of several sharp diffraction peaks. Apart from a few amorphous minerals (coals), numerous high-density minerals such as quartz, kaolinite, and pyrite are enriched in the heavy product.

**Figure 6:**
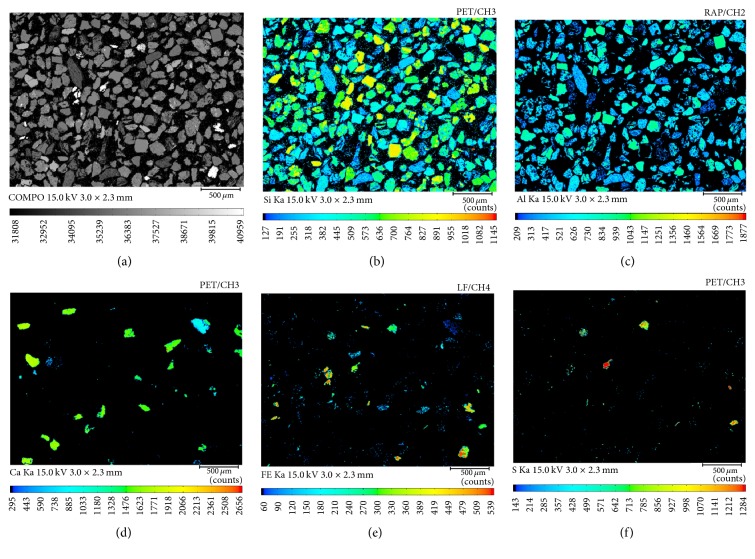
Surface distribution of elements in heavy product: (a) backscatter imagery of heavy product; (b) Si; (c) Al; (d) Ca; (e) Fe; (f) S.

The backscatter imagery and surface distribution of elements in heavy product are shown in Figure 6(). In the backscatter imagery, high-density particles with high average atomic number are bright, especially the particles that contain iron. The main elements in the heavy product are Si, Al, and Ca. In other words, aluminosilicate minerals are the main minerals in the heavy product. In addition, sulfur and iron exist in the heavy product. The distribution of sulfur is also the same as that of iron, thereby indicating that S exists in the form of pyrite. Therefore, pyrite has been separated during the separation experiment.

**Figure 7:**
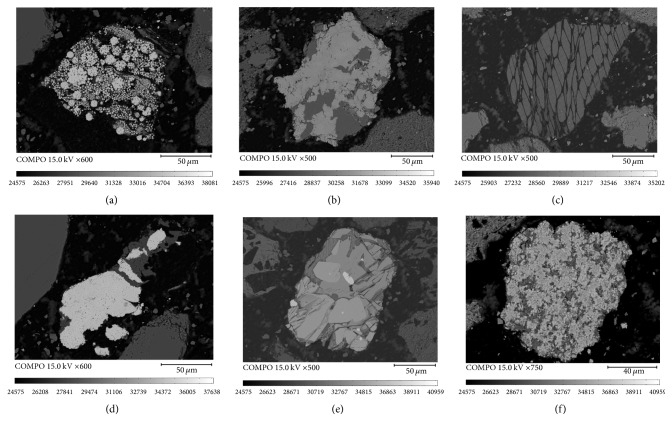
Backscatter imagery of different mineral in heavy product: (a) pyrite; (b) quartz; (c) kaolinite; (d) iron oxide; (e) chalcopyrite; (f) gypsum.

The backscatter imagery of different minerals in heavy product by qualitative analysis is shown in Figure 7(). Obviously, high-density minerals such as pyrite, quartz, kaolinite, chalcopyrite, gypsum, and iron oxide are effectively separated in the separation process.

**Figure 8:**
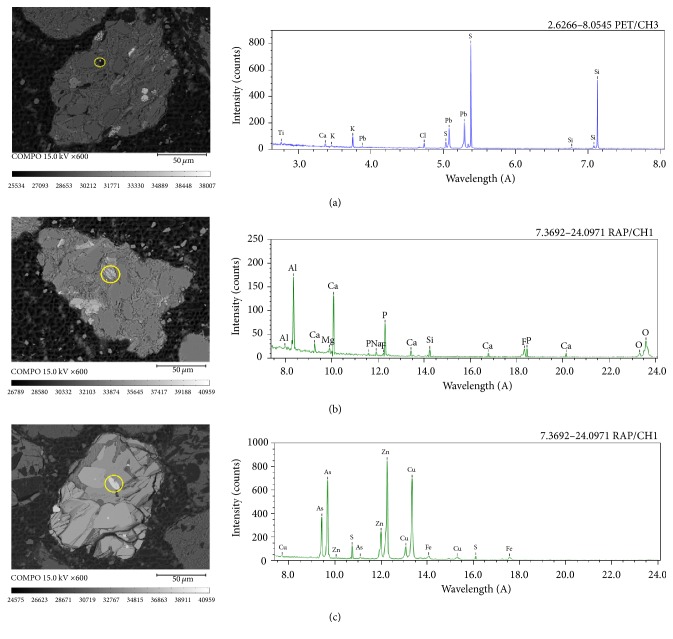
Qualitative analysis of particles containing harmful elements: (a) lead particle; (b) fluoride particle; (c) arsenic particle.

Some harmful elements such as Pb, As, and F are also found in the heavy product by qualitative analysis as shown in Figure 8(). These particles contain harmful elements that are generally distributed in large particles with tiny sizes. In addition, S and Fe always appear in the same particle containing the abovementioned harmful elements. This condition means that pyrite is the important medium for those elements. Thus, removal of pyrite is the key to the desulfurization and detoxification of fine coal.

## 4. Conclusion

The 0.5–0.125 mm fraction fine coal is separated effectively by ultrasonic vibration gas-solid fluidized bed. After adding the ultrasonic vibration force field, the particle separation process based on density is strengthened, and the best result appears when the vibration frequency is 35 kHz. The ash difference between the light and heavy products and the recovery of combustible material reach the maximum values of 47.07% and 89.04%, respectively. Airflow velocity significantly influences the separation. When the fluidization number is low, the bed liquidity is poor and the resistance to particle sedimentation is high. Conversely, the bed stability is destroyed and the back mixing between the light and heavy products occurs, thereby hindering the separation. When the vibration frequency is 35 kHz and the fluidization number is 1.8, the ash difference between the light and heavy products and the recovery of combustible material obtain the maximum values of 47.30% and 89.59%, respectively. Sulfur is mainly concentrated in the heavy product, and the content reaches the maximum value of 6.78%.

The XPS results show that -C-S-, -SO_4_, and -S_2_ exist in the original coal and heavy product; however, the content of -S_2_ in the heavy product is higher than that in the original coal. On the contrary, -C-S- mainly exists in the light product. According to the peak area, pyrite and sulfate are evidently enriched in the heavy product. The XRF and XRD results also show that several high-density minerals such as quartz, kaolinite, and pyrite are enriched in the heavy product. Some harmful elements such as F, Pb, and As are also found in the heavy product by EPMA. These elements are generally distributed in large particles with tiny sizes. Therefore, fine coal is effectively separated by the proposed method and thus the target of desulfurization and deashing by ultrasonic vibration gas-solid fluidized bed is realized.

## Figures and Tables

**Table 1:** Sulfur content of each layer of product.

Layer number	Sulfur content/%					
	0 Hz	20 kHz	25 kHz	30 kHz	35 kHz	40 kHz
First layer	1.52	1.38	1.35	1.33	1.26	1.48
Second layer	2.11	1.96	1.93	1.86	1.82	1.98
Third layer	2.23	2.18	2.26	2.14	2.22	2.08
Forth layer	2.26	2.32	2.35	2.28	2.25	2.29
Fifth layer	6.56	6.75	6.73	6.68	6.89	6.77

**Table 2:** Sulfur content of each layer of product.

Layer number	Sulfur content/%						
	1.2	1.4	1.6	1.8	2.0	2.2	2.4
First layer	2.12	1.76	1.34	1.25	1.22	1.58	2.25
Second layer	2.23	2.03	1.92	1.78	1.80	1.89	2.28
Third layer	2.33	2.38	2.02	1.84	1.92	2.44	2.96
Forth layer	2.45	2.27	2.13	2.10	2.15	2.53	3.12
Fifth layer	5.36	6.15	6.33	6.78	6.59	5.62	4.98

**Table 3:** Elemental composition of different samples.

Sample name	Element content/%						
	MgO	CaO	Fe_2_O_3_	Al_2_O_3_	SiO_2_	S	P
Original coal	0.16	2.74	2.02	11.91	13.06	2.43	0.021
Light product	0.10	0.61	0.63	9.76	12.07	1.66	0.022
Heavy product	0.58	2.03	3.68	20.38	39.28	6.78	0.029

**Table 4:** Content of sulfur in different chemical states.

Name	Original coal		Heavy product	
	Peak BE	Atomic%	Peak BE	Atomic%
-SO_4_	168.91	40.75	168.88	24.03
-C-S-	164.06	45.75	163.97	23.43
-S_2_	162.78	13.5	162.7	52.54
